# Supplementary material for: The cumulative disadvantage of unemployment: Longitudinal evidence across gender and age at first unemployment in Germany
Source: PLoS One. 2020 Jun 24;15(6):e0234786. doi: 10.1371/journal.pone.0234786 (PMC7313743; doi:10.1371/journal.pone.0234786)
Supplement: S1 Table — Men and Women. (DOCX) [file pone.0234786.s001.docx]

**S1 Table. Hybrid model results. Men and Women.**

|  | Men | Women | Significance of gender difference |
| --- | --- | --- | --- |
| *Time since unemployment* |  |  |  |
| first month in unemployment | -0.758*** | -0.609*** | *** |
| Trimester 1 | -0.607*** | -0.492*** | *** |
| Trimester 2 | -0.463*** | -0.398*** | * |
| Trimester 3 | -0.392*** | -0.345*** |  |
| Trimester 4 | -0.349*** | -0.311*** |  |
| Trimester 5 | -0.329*** | -0.287*** |  |
| Trimester 6 | -0.303*** | -0.266*** |  |
| Trimester 7 | -0.282*** | -0.247*** |  |
| Trimester 8 | -0.266*** | -0.232*** |  |
| Trimester 9 | -0.254*** | -0.221*** |  |
| Trimester 10 | -0.246*** | -0.214*** |  |
| Trimester 11 | -0.241*** | -0.208*** |  |
| Trimester 12 | -0.237*** | -0.206*** |  |
| Trimester 13 | -0.234*** | -0.202*** |  |
| Trimester 14 | -0.229*** | -0.198*** |  |
| Trimester 15 | -0.224*** | -0.195*** |  |
| Trimester 16 | -0.221*** | -0.192*** |  |
| Trimester 17 | -0.218*** | -0.192*** |  |
| Trimester 18 | -0.212*** | -0.192*** |  |
| Trimester 19 | -0.206*** | -0.192*** |  |
| Trimester 20 | -0.202*** | -0.189*** |  |
| Trimester 21 | -0.198*** | -0.187*** |  |
| Trimester 22 | -0.191*** | -0.185*** |  |
| Trimester 23 | -0.188*** | -0.185*** |  |
| Trimester 24 | -0.184*** | -0.185*** |  |
| Trimester 25 | -0.179*** | -0.184*** |  |
| Trimester 26 | -0.176*** | -0.186*** |  |
| Trimester 27 | -0.177*** | -0.189*** |  |
| Trimester 28 | -0.175*** | -0.190*** |  |
| Trimester 29 | -0.174*** | -0.192*** |  |
| Trimester 30 | -0.173*** | -0.195*** |  |
| Trimester 31 | -0.172*** | -0.198*** |  |
| Trimester 32 | -0.168*** | -0.200*** |  |
| Trimester 33 | -0.163*** | -0.204*** |  |
| Trimester 34 | -0.158*** | -0.207*** |  |
| Trimester 35 | -0.154** | -0.210*** |  |
| Trimester 36 | -0.151** | -0.211*** |  |
| Trimester 37 | -0.143** | -0.214*** |  |
| Trimester 38 | -0.135** | -0.219*** |  |
| Trimester 39 | -0.132* | -0.221*** |  |
| Trimester 40 | -0.130* | -0.224*** |  |
| Trimester 41 | -0.131* | -0.228*** |  |
| Trimester 42 | -0.134* | -0.233*** |  |
| Trimester 43 | -0.134* | -0.243*** |  |
| Trimester 44 | -0.133* | -0.245*** |  |
| Trimester 45 | -0.132* | -0.245*** |  |
| Trimester 46 | -0.137* | -0.253*** |  |
| Trimester 47 | -0.140* | -0.256*** |  |
| Trimester 48 | -0.139* | -0.257*** |  |
| Trimester 49 | -0.140* | -0.260*** |  |
| Trimester 50 | -0.142* | -0.266*** |  |
| Trimester 51 | -0.138* | -0.275*** |  |
| Trimester 52 | -0.139* | -0.276*** |  |
| Trimester 53 | -0.145* | -0.280*** |  |
| Trimester 54 | -0.145* | -0.280*** |  |
| Trimester 55 | -0.146* | -0.280*** |  |
| Trimester 56 | -0.144* | -0.277*** |  |
| Trimester 57 | -0.142* | -0.274*** |  |
| Trimester 58 | -0.138 | -0.276*** |  |
| Trimester 59 | -0.137 | -0.276*** |  |
| Trimester 60 | -0.134 | -0.276*** |  |
| Trimester 61 | -0.134 | -0.277*** |  |
| Trimester 62 | -0.131 | -0.276** |  |
| Trimester 63 | -0.126 | -0.288*** |  |
| Trimester 64 | -0.125 | -0.289*** |  |
| Trimester 65 | -0.125 | -0.291*** |  |
| Trimester 66 | -0.122 | -0.295*** |  |
| Trimester 67 | -0.121 | -0.316*** |  |
| Trimester 68 | -0.118 | -0.320*** |  |
| Trimester 69 | -0.118 | -0.322*** |  |
| Trimester 70 | -0.121 | -0.323*** |  |
| Trimester 71 | -0.119 | -0.326*** |  |
| Trimester 72 | -0.112 | -0.323*** |  |
| Trimester 73 | -0.11 | -0.326*** |  |
| Trimester 74 | -0.0994 | -0.332*** |  |
| Trimester 75 | -0.0872 | -0.327** |  |
| Trimester 76 | -0.0857 | -0.315** |  |
| Trimester 77 | -0.0847 | -0.315** |  |
| Trimester 78 | -0.0845 | -0.313** |  |
| Trimester 79 | -0.0867 | -0.296** |  |
| Trimester 80 | -0.0954 | -0.276* |  |
| Trimester 81 | -0.0942 | -0.278* |  |
| Trimester 82 | -0.0995 | -0.284* |  |
| Trimester 83 | -0.104 | -0.294* |  |
| Trimester 84 | -0.109 | -0.287* |  |
| Trimester 85 | -0.101 | -0.262* |  |
| Trimester 86 | -0.0983 | -0.275* |  |
| Trimester 87 | -0.0849 | -0.252* |  |
| Trimester 88 | -0.0607 | -0.239 |  |
| Trimester 89 | -0.127 | -0.207 |  |
| Trimester 90 |  | -0.244* |  |
| Age | 0.00352*** | 0.000999 | *** |
| Age squared | -0.000004*** | 0.0000001 |  |
| *Education (ref: low)* |  |  |  |
| Low intermediate | 0.0639 | 0.0883* |  |
| High intermediate | -0.0634 | 0.00168 |  |
| High | 0.0897 | 0.149 |  |
| Career quality before unemployment | 0.176*** | 0.232*** |  |
| GDP | 0.00751* | 0.00176 |  |
| Constant | -0.938 | 0.682** |  |
| N | 41,677 | 51,677 |  |
