# Supplementary material for: The cumulative disadvantage of unemployment: Longitudinal evidence across gender and age at first unemployment in Germany
Source: PLoS One. 2020 Jun 24;15(6):e0234786. doi: 10.1371/journal.pone.0234786 (PMC7313743; doi:10.1371/journal.pone.0234786)
Supplement: S2 Table — (DOCX) [file pone.0234786.s002.docx]

**S2 Table. Hybrid model results: Subsamples of Men and Women ages 25-35 and 36-45.**

|  | Childbearing ages (25-35) | | | Childrearing ages (36-45) | | |
| --- | --- | --- | --- | --- | --- | --- |
|  | MEN | WOMEN | Signif. of gender diff | MEN | WOMEN | Signif. of gender diff |
| *Time since unemployment* | |  |  |  |  |  |
| first month in unemployment | -0.774*** | -0.549*** | *** | -0.844*** | -0.594*** | *** |
| Trimester 1 | -0.550*** | -0.433*** | * | -0.663*** | -0.499*** | * |
| Trimester 2 | -0.341*** | -0.356*** |  | -0.485*** | -0.413*** |  |
| Trimester 3 | -0.236*** | -0.315*** |  | -0.408*** | -0.358*** |  |
| Trimester 4 | -0.165*** | -0.288*** |  | -0.372*** | -0.308*** |  |
| Trimester 5 | -0.124** | -0.268*** | * | -0.343*** | -0.277*** |  |
| Trimester 6 | -0.0875 | -0.257*** | * | -0.315*** | -0.253*** |  |
| Trimester 7 | -0.0661 | -0.249*** | ** | -0.299*** | -0.234*** |  |
| Trimester 8 | -0.056 | -0.239*** | ** | -0.281*** | -0.224*** |  |
| Trimester 9 | -0.0571 | -0.233*** | * | -0.271*** | -0.215*** |  |
| Trimester 10 | -0.0574 | -0.230*** | * | -0.279*** | -0.210*** |  |
| Trimester 11 | -0.0634 | -0.219*** |  | -0.292*** | -0.213*** |  |
| Trimester 12 | -0.0684 | -0.210*** |  | -0.304*** | -0.223*** |  |
| Trimester 13 | -0.0689 | -0.205*** |  | -0.313*** | -0.229*** |  |
| Trimester 14 | -0.0691 | -0.205*** |  | -0.332*** | -0.235*** |  |
| Trimester 15 | -0.066 | -0.206*** |  | -0.351*** | -0.245*** |  |
| Trimester 16 | -0.0628 | -0.206*** |  | -0.379*** | -0.253*** |  |
| Trimester 17 | -0.0607 | -0.207*** |  | -0.397*** | -0.267*** |  |
| Trimester 18 | -0.0598 | -0.213*** |  | -0.411*** | -0.276*** |  |
| Trimester 19 | -0.056 | -0.217*** |  | -0.417*** | -0.276*** |  |
| Trimester 20 | -0.0554 | -0.221*** |  | -0.427*** | -0.276*** |  |
| Trimester 21 | -0.0575 | -0.224*** |  | -0.439*** | -0.277** |  |
| Trimester 22 | -0.0567 | -0.228*** |  | -0.442*** | -0.277** |  |
| Trimester 23 | -0.0646 | -0.228*** |  | -0.456*** | -0.285** |  |
| Trimester 24 | -0.0749 | -0.231*** |  | -0.473*** | -0.293** |  |
| Trimester 25 | -0.079 | -0.236*** |  | -0.484*** | -0.292** |  |
| Trimester 26 | -0.0846 | -0.244*** |  | -0.487*** | -0.296** |  |
| Trimester 27 | -0.0914 | -0.251*** |  | -0.494*** | -0.313** |  |
| Trimester 28 | -0.0984 | -0.252*** |  | -0.500*** | -0.317** |  |
| Trimester 29 | -0.102 | -0.255*** |  | -0.500*** | -0.328** |  |
| Trimester 30 | -0.106 | -0.259*** |  | -0.510*** | -0.338** |  |
| Trimester 31 | -0.111 | -0.265*** |  | -0.521*** | -0.347** |  |
| Trimester 32 | -0.115 | -0.274*** |  | -0.528*** | -0.353** |  |
| Trimester 33 | -0.118 | -0.284*** |  | -0.536*** | -0.354** |  |
| Trimester 34 | -0.118 | -0.295*** |  | -0.538*** | -0.361** |  |
| Trimester 35 | -0.121 | -0.302*** |  | -0.541*** | -0.367** |  |
| Trimester 36 | -0.116 | -0.305*** |  | -0.550*** | -0.375** |  |
| Trimester 37 | -0.113 | -0.314*** |  | -0.558*** | -0.382** |  |
| Trimester 38 | -0.112 | -0.329*** |  | -0.566*** | -0.388** |  |
| Trimester 39 | -0.113 | -0.337*** |  | -0.576*** | -0.396** |  |
| Trimester 40 | -0.115 | -0.345*** |  | -0.584*** | -0.402** |  |
| Trimester 41 | -0.121 | -0.347*** |  | -0.596*** | -0.413** |  |
| Trimester 42 | -0.126 | -0.356*** |  | -0.637*** | -0.431** |  |
| Trimester 43 | -0.131 | -0.369*** |  | -0.653*** | -0.461** |  |
| Trimester 44 | -0.138 | -0.378*** |  | -0.671*** | -0.464** |  |
| Trimester 45 | -0.144 | -0.387*** |  | -0.688*** | -0.461** |  |
| Trimester 46 | -0.16 | -0.395*** |  | -0.707*** | -0.474** |  |
| Trimester 47 | -0.17 | -0.400*** |  | -0.736*** | -0.484** |  |
| Trimester 48 | -0.177 | -0.405*** |  | -0.752*** | -0.491** |  |
| Trimester 49 | -0.189 | -0.410*** |  | -0.768*** | -0.500** |  |
| Trimester 50 | -0.195 | -0.421*** |  | -0.783*** | -0.505** |  |
| Trimester 51 | -0.196 | -0.444*** |  | -0.799*** | -0.511** |  |
| Trimester 52 | -0.201 | -0.457*** |  | -0.817*** | -0.518** |  |
| Trimester 53 | -0.211 | -0.473*** |  | -0.836*** | -0.519** |  |
| Trimester 54 | -0.216 | -0.483*** |  | -0.855*** | -0.521** |  |
| Trimester 55 | -0.219 | -0.491*** |  | -0.873*** | -0.523** |  |
| Trimester 56 | -0.223 | -0.497*** |  | -0.887*** | -0.528** |  |
| Trimester 57 | -0.227 | -0.502*** |  | -0.902*** | -0.536** |  |
| Trimester 58 | -0.231 | -0.514*** |  | -0.895*** | -0.538* |  |
| Trimester 59 | -0.234 | -0.523*** |  | -0.916*** | -0.539* |  |
| Trimester 60 | -0.237 | -0.530*** |  | -0.932*** | -0.540* |  |
| Trimester 61 | -0.247 | -0.540*** |  | -0.940*** | -0.542* |  |
| Trimester 62 | -0.255 | -0.542*** |  | -0.946*** | -0.548* |  |
| Trimester 63 | -0.262 | -0.557*** |  | -0.934*** | -0.563* |  |
| Trimester 64 | -0.269 | -0.566*** |  | -0.951*** | -0.564* |  |
| Trimester 65 | -0.276 | -0.573*** |  | -0.968*** | -0.573* |  |
| Trimester 66 | -0.277 | -0.583*** |  | -0.985*** | -0.583* |  |
| Trimester 67 | -0.281 | -0.622*** |  | -1.002*** | -0.591* |  |
| Trimester 68 | -0.287 | -0.640*** |  | -1.022*** | -0.599* |  |
| Trimester 69 | -0.291 | -0.651*** |  | -1.043*** | -0.605* |  |
| Trimester 70 | -0.289 | -0.661*** |  | -1.064*** | -0.612* |  |
| Trimester 71 | -0.289 | -0.671*** |  | -1.084*** | -0.617* |  |
| Trimester 72 | -0.295 | -0.681*** |  | -1.105*** | -0.621* |  |
| Trimester 73 | -0.3 | -0.692*** |  | -1.124*** | -0.623* |  |
| Trimester 74 | -0.298 | -0.702*** |  | -1.167*** | -0.624* |  |
| Trimester 75 | -0.27 | -0.706*** |  | -1.191*** | -0.622* |  |
| Trimester 76 | -0.271 | -0.687*** |  | -1.216*** | -0.629* |  |
| Trimester 77 | -0.276 | -0.664** |  | -1.241*** | -0.647* |  |
| Trimester 78 | -0.283 | -0.676** |  | -1.272*** | -0.649* |  |
| Trimester 79 | -0.291 | -0.664** |  | -1.305*** | -0.647* |  |
| Trimester 80 | -0.299 | -0.653** |  | -1.341*** | -0.605 |  |
| Trimester 81 | -0.309 | -0.665** |  | -1.368*** | -0.583 |  |
| Trimester 82 | -0.319 | -0.675** |  | -1.469*** | -0.581 |  |
| Trimester 83 | -0.33 | -0.703** |  | -1.584*** | -0.642 |  |
| Trimester 84 | -0.366 | -0.712** |  | -1.619*** | -0.628 |  |
| Trimester 85 | -0.371 | -0.721** |  | -1.654*** | -0.546 |  |
| Trimester 86 | -0.374 | -0.782*** |  | -1.688*** | -0.546 |  |
| Trimester 87 | -0.374 | -0.778** |  | -1.721*** | . |  |
| Trimester 88 | -0.348 | -0.806** |  |  |  |  |
| Trimester 89 | -0.414 | -1.013** |  |  |  |  |
| Trimester 90 |  |  |  |  |  |  |
| Age | 0.00186 | -0.00301 |  | -0.000526 | 0.0208*** | ** |
| Age squared | 0.0000002 | 0.000007 |  | 0.000004 | -0.00002*** | ** |
| *Education (ref: low)* | |  |  |  |  |  |
| Low intermediate | 0.159* | 0.0829 |  | -0.0346 | 0.0672 |  |
| High intermediate | 0.0507 | -4.382 |  | . | -0.089 |  |
| High | 0.0504 | -3.732 |  | -1.111 | -0.0206 |  |
| Career quality before unemployment | 0.0283 | 0.257*** | * | 0.474*** | 0.167 | * |
| GDP | 0.00888 | -0.00344 |  | 0.00103 | -0.00556 |  |
| Constant | 0.981 | -38.13*** |  | -4.156 | -18.44*** |  |
| N | 11,901 | 19,233 |  | 5,469 | 8,639 |  |
