# Supplementary material for: The cumulative disadvantage of unemployment: Longitudinal evidence across gender and age at first unemployment in Germany
Source: PLoS One. 2020 Jun 24;15(6):e0234786. doi: 10.1371/journal.pone.0234786 (PMC7313743; doi:10.1371/journal.pone.0234786)
Supplement: S3 Table — A. Hybrid model results: Men experiencing unemployment at different ages. B. Hybrid model results: Women experiencing unemployment at different ages. (DOCX) [file pone.0234786.s003.docx]

**S3A Table. Hybrid model results: Men experiencing unemployment at different ages.**

|  | MEN | | | | |
| --- | --- | --- | --- | --- | --- |
|  | age 18-24 | age 25-35 | age 36-45 | age 46-54 | age 55-64 |
| *Time since unemployment* |  |  |  |  |  |
| first month in unemployment | -0.582*** | -0.774*** | -0.844*** | -0.859*** | -0.904*** |
| Trimester 1 | -0.399*** | -0.550*** | -0.663*** | -0.782*** | -0.891*** |
| Trimester 2 | -0.235*** | -0.341*** | -0.485*** | -0.708*** | -0.878*** |
| Trimester 3 | -0.167*** | -0.236*** | -0.408*** | -0.670*** | -0.867*** |
| Trimester 4 | -0.141** | -0.165*** | -0.372*** | -0.637*** | -0.859*** |
| Trimester 5 | -0.153*** | -0.124** | -0.343*** | -0.635*** | -0.850*** |
| Trimester 6 | -0.153*** | -0.0875 | -0.315*** | -0.615*** | -0.839*** |
| Trimester 7 | -0.151*** | -0.0661 | -0.299*** | -0.590*** | -0.833*** |
| Trimester 8 | -0.153** | -0.056 | -0.281*** | -0.582*** | -0.828*** |
| Trimester 9 | -0.153** | -0.0571 | -0.271*** | -0.564*** | -0.830*** |
| Trimester 10 | -0.154** | -0.0574 | -0.279*** | -0.560*** | -0.833*** |
| Trimester 11 | -0.164*** | -0.0634 | -0.292*** | -0.557*** | -0.829*** |
| Trimester 12 | -0.175*** | -0.0684 | -0.304*** | -0.553*** | -0.826*** |
| Trimester 13 | -0.189*** | -0.0689 | -0.313*** | -0.557*** | -0.824*** |
| Trimester 14 | -0.201*** | -0.0691 | -0.332*** | -0.559*** | -0.819*** |
| Trimester 15 | -0.216*** | -0.066 | -0.351*** | -0.561*** | -0.813*** |
| Trimester 16 | -0.233*** | -0.0628 | -0.379*** | -0.564*** | -0.810*** |
| Trimester 17 | -0.253*** | -0.0607 | -0.397*** | -0.566*** | -0.807*** |
| Trimester 18 | -0.267*** | -0.0598 | -0.411*** | -0.567*** | -0.801*** |
| Trimester 19 | -0.280*** | -0.056 | -0.417*** | -0.579*** | -0.793*** |
| Trimester 20 | -0.297*** | -0.0554 | -0.427*** | -0.589*** | -0.786*** |
| Trimester 21 | -0.311*** | -0.0575 | -0.439*** | -0.596*** | -0.779*** |
| Trimester 22 | -0.326*** | -0.0567 | -0.442*** | -0.599*** | -0.774*** |
| Trimester 23 | -0.339*** | -0.0646 | -0.456*** | -0.599*** | -0.769*** |
| Trimester 24 | -0.353*** | -0.0749 | -0.473*** | -0.596*** | -0.764*** |
| Trimester 25 | -0.367*** | -0.079 | -0.484*** | -0.599*** | -0.761*** |
| Trimester 26 | -0.386*** | -0.0846 | -0.487*** | -0.607*** | -0.756*** |
| Trimester 27 | -0.413*** | -0.0914 | -0.494*** | -0.624*** | -0.751*** |
| Trimester 28 | -0.430*** | -0.0984 | -0.500*** | -0.637*** | -0.754*** |
| Trimester 29 | -0.451*** | -0.102 | -0.500*** | -0.645*** | -0.770*** |
| Trimester 30 | -0.475*** | -0.106 | -0.510*** | -0.652*** | -0.769*** |
| Trimester 31 | -0.500*** | -0.111 | -0.521*** | -0.658*** | -0.763*** |
| Trimester 32 | -0.521*** | -0.115 | -0.528*** | -0.662*** | -0.756*** |
| Trimester 33 | -0.536*** | -0.118 | -0.536*** | -0.674*** | -0.748*** |
| Trimester 34 | -0.549*** | -0.118 | -0.538*** | -0.694*** | -0.745*** |
| Trimester 35 | -0.571*** | -0.121 | -0.541*** | -0.700*** | -0.739*** |
| Trimester 36 | -0.591*** | -0.116 | -0.550*** | -0.706*** | -0.870*** |
| Trimester 37 | -0.611*** | -0.113 | -0.558*** | -0.710*** | -0.863*** |
| Trimester 38 | -0.635*** | -0.112 | -0.566*** | -0.709*** | -0.755*** |
| Trimester 39 | -0.661*** | -0.113 | -0.576*** | -0.714*** | -0.751*** |
| Trimester 40 | -0.689*** | -0.115 | -0.584*** | -0.716*** | -0.748*** |
| Trimester 41 | -0.722*** | -0.121 | -0.596*** | -0.726*** | . |
| Trimester 42 | -0.747*** | -0.126 | -0.637*** | -0.731*** | . |
| Trimester 43 | -0.774*** | -0.131 | -0.653*** | -0.740*** | . |
| Trimester 44 | -0.799*** | -0.138 | -0.671*** | -0.754*** | . |
| Trimester 45 | -0.822*** | -0.144 | -0.688*** | -0.769*** | . |
| Trimester 46 | -0.848*** | -0.16 | -0.707*** | -0.803*** | . |
| Trimester 47 | -0.874*** | -0.17 | -0.736*** | -0.816*** | . |
| Trimester 48 | -0.899*** | -0.177 | -0.752*** | -0.822*** | . |
| Trimester 49 | -0.927*** | -0.189 | -0.768*** | -0.823*** | . |
| Trimester 50 | -0.964*** | -0.195 | -0.783*** | -0.821*** | . |
| Trimester 51 | -0.990*** | -0.196 | -0.799*** | -0.831*** | . |
| Trimester 52 | -1.019*** | -0.201 | -0.817*** | -0.849*** | . |
| Trimester 53 | -1.056*** | -0.211 | -0.836*** | -0.868*** | . |
| Trimester 54 | -1.084*** | -0.216 | -0.855*** | -0.885*** | . |
| Trimester 55 | -1.114*** | -0.219 | -0.873*** | -0.903*** | . |
| Trimester 56 | -1.136*** | -0.223 | -0.887*** | -0.896*** | . |
| Trimester 57 | -1.160*** | -0.227 | -0.902*** | -0.902*** | . |
| Trimester 58 | -1.191*** | -0.231 | -0.895*** | -0.923*** | . |
| Trimester 59 | -1.219*** | -0.234 | -0.916*** | -0.943*** | . |
| Trimester 60 | -1.247*** | -0.237 | -0.932*** | -0.964*** | . |
| Trimester 61 | -1.273*** | -0.247 | -0.940*** | -0.980*** | . |
| Trimester 62 | -1.299*** | -0.255 | -0.946*** | -0.996*** | . |
| Trimester 63 | -1.327*** | -0.262 | -0.934*** | -1.010*** | . |
| Trimester 64 | -1.355*** | -0.269 | -0.951*** | -1.023*** | . |
| Trimester 65 | -1.384*** | -0.276 | -0.968*** | -1.036*** | . |
| Trimester 66 | -1.419*** | -0.277 | -0.985*** | -1.047*** | . |
| Trimester 67 | -1.452*** | -0.281 | -1.002*** | -1.057*** | . |
| Trimester 68 | -1.482*** | -0.287 | -1.022*** | -1.067*** | . |
| Trimester 69 | -1.518*** | -0.291 | -1.043*** | -1.078*** | . |
| Trimester 70 | -1.560*** | -0.289 | -1.064*** | -1.152*** | . |
| Trimester 71 | -1.591*** | -0.289 | -1.084*** | -1.216*** | . |
| Trimester 72 | -1.621*** | -0.295 | -1.105*** | -1.235*** | . |
| Trimester 73 | -1.666*** | -0.3 | -1.124*** | . | . |
| Trimester 74 | -1.679*** | -0.298 | -1.167*** | . | . |
| Trimester 75 | -1.711*** | -0.27 | -1.191*** | . | . |
| Trimester 76 | -1.746*** | -0.271 | -1.216*** | . | . |
| Trimester 77 | -1.780*** | -0.276 | -1.241*** | . | . |
| Trimester 78 | -1.814*** | -0.283 | -1.272*** | . | . |
| Trimester 79 | -1.870*** | -0.291 | -1.305*** | . | . |
| Trimester 80 | -1.898*** | -0.299 | -1.341*** | . | . |
| Trimester 81 | -1.938*** | -0.309 | -1.368*** | . | . |
| Trimester 82 | -1.977*** | -0.319 | -1.469*** | . | . |
| Trimester 83 | -2.013*** | -0.33 | -1.584*** | . | . |
| Trimester 84 | -2.047*** | -0.366 | -1.619*** | . | . |
| Trimester 85 | -2.065*** | -0.371 | -1.654*** | . | . |
| Trimester 86 | -2.113*** | -0.374 | -1.688*** | . | . |
| Trimester 87 | -2.132*** | -0.374 | -1.721*** | . | . |
| Trimester 88 | . | -0.348 | . | . | . |
| Trimester 89 | . | -0.414 | . | . | . |
| Age | -0.000471 | 0.00186 | -0.000526 | 0.0244*** | 0.0147 |
| Age squared | 0.000012* | 0.0000002 | 0.0000042 | -0.000017** | -0.000011 |
| *Education (ref: low)* |  |  |  |  |  |
| Low intermediate | -0.0415 | 0.159* | -0.0346 | 0.186 | 0.00734 |
| High intermediate | -0.180* | 0.0507 | . | . | -0.0269 |
| High | 0.0146 | 0.0504 | -1.111 | -0.0358 | . |
| Career quality before unemployment | 0.206*** | 0.0283 | 0.474*** | 0.597*** | 0.104 |
| GDP | 0.00411 | 0.00888 | 0.00103 | 0.00287 | -0.000309 |
| Constant | 3.632* | 0.981 | -4.156 | -18.80*** | -6.32 |
| Observations | 13,436 | 11,901 | 5,469 | 4,546 | 6,325 |

Note: grayed cells indicate that effects are significantly different (p<0.05) compared to the reference category. This was tested in a model including interactions between each age group and time, with the youngest age group as reference category.

**S3B Table. Hybrid model results: Women experiencing unemployment at different ages.**

|  | WOMEN | | | | |
| --- | --- | --- | --- | --- | --- |
|  | age 18-24 | age 25-35 | age 36-45 | age 46-54 | age 55-64 |
| *Time since unemployment* |  |  |  |  |  |
| first month in unemployment | -0.655*** | -0.549*** | -0.594*** | -0.706*** | -0.629*** |
| Trimester 1 | -0.486*** | -0.433*** | -0.499*** | -0.642*** | -0.615*** |
| Trimester 2 | -0.349*** | -0.356*** | -0.413*** | -0.599*** | -0.592*** |
| Trimester 3 | -0.279*** | -0.315*** | -0.358*** | -0.572*** | -0.590*** |
| Trimester 4 | -0.243*** | -0.288*** | -0.308*** | -0.557*** | -0.592*** |
| Trimester 5 | -0.220*** | -0.268*** | -0.277*** | -0.555*** | -0.597*** |
| Trimester 6 | -0.195*** | -0.257*** | -0.253*** | -0.538*** | -0.593*** |
| Trimester 7 | -0.175** | -0.249*** | -0.234*** | -0.517*** | -0.588*** |
| Trimester 8 | -0.164** | -0.239*** | -0.224*** | -0.499*** | -0.587*** |
| Trimester 9 | -0.160** | -0.233*** | -0.215*** | -0.487*** | -0.587*** |
| Trimester 10 | -0.161** | -0.230*** | -0.210*** | -0.475*** | -0.588*** |
| Trimester 11 | -0.167** | -0.219*** | -0.213*** | -0.474*** | -0.593*** |
| Trimester 12 | -0.180** | -0.210*** | -0.223*** | -0.472*** | -0.599*** |
| Trimester 13 | -0.186** | -0.205*** | -0.229*** | -0.480*** | -0.606*** |
| Trimester 14 | -0.187** | -0.205*** | -0.235*** | -0.479*** | -0.610*** |
| Trimester 15 | -0.186** | -0.206*** | -0.245*** | -0.480*** | -0.617*** |
| Trimester 16 | -0.188* | -0.206*** | -0.253*** | -0.477*** | -0.623*** |
| Trimester 17 | -0.194** | -0.207*** | -0.267*** | -0.480*** | -0.630*** |
| Trimester 18 | -0.198* | -0.213*** | -0.276*** | -0.490*** | -0.633*** |
| Trimester 19 | -0.204* | -0.217*** | -0.276*** | -0.499*** | -0.638*** |
| Trimester 20 | -0.211** | -0.221*** | -0.276*** | -0.494*** | -0.645*** |
| Trimester 21 | -0.217** | -0.224*** | -0.277** | -0.495*** | -0.649*** |
| Trimester 22 | -0.223** | -0.228*** | -0.277** | -0.499*** | -0.649*** |
| Trimester 23 | -0.229** | -0.228*** | -0.285** | -0.521*** | -0.650*** |
| Trimester 24 | -0.238** | -0.231*** | -0.293** | -0.529*** | -0.652*** |
| Trimester 25 | -0.244** | -0.236*** | -0.292** | -0.534*** | -0.655*** |
| Trimester 26 | -0.258** | -0.244*** | -0.296** | -0.543*** | -0.658*** |
| Trimester 27 | -0.269** | -0.251*** | -0.313** | -0.556*** | -0.658*** |
| Trimester 28 | -0.287** | -0.252*** | -0.317** | -0.575*** | -0.660*** |
| Trimester 29 | -0.307** | -0.255*** | -0.328** | -0.583*** | -0.665*** |
| Trimester 30 | -0.327** | -0.259*** | -0.338** | -0.591*** | -0.668*** |
| Trimester 31 | -0.347*** | -0.265*** | -0.347** | -0.600*** | -0.661*** |
| Trimester 32 | -0.365*** | -0.274*** | -0.353** | -0.608*** | -0.654*** |
| Trimester 33 | -0.383*** | -0.284*** | -0.354** | -0.613*** | -0.641*** |
| Trimester 34 | -0.397*** | -0.295*** | -0.361** | -0.614*** | -0.587*** |
| Trimester 35 | -0.414*** | -0.302*** | -0.367** | -0.621*** | -0.539*** |
| Trimester 36 | -0.429*** | -0.305*** | -0.375** | -0.629*** | . |
| Trimester 37 | -0.445*** | -0.314*** | -0.382** | -0.637*** | . |
| Trimester 38 | -0.461*** | -0.329*** | -0.388** | -0.641*** | . |
| Trimester 39 | -0.479*** | -0.337*** | -0.396** | -0.646*** | . |
| Trimester 40 | -0.503*** | -0.345*** | -0.402** | -0.651*** | . |
| Trimester 41 | -0.524*** | -0.347*** | -0.413** | -0.660*** | . |
| Trimester 42 | -0.544*** | -0.356*** | -0.431** | -0.665*** | . |
| Trimester 43 | -0.561*** | -0.369*** | -0.461** | -0.670*** | . |
| Trimester 44 | -0.574*** | -0.378*** | -0.464** | -0.680*** | . |
| Trimester 45 | -0.585*** | -0.387*** | -0.461** | -0.670*** | . |
| Trimester 46 | -0.611*** | -0.395*** | -0.474** | -0.685*** | . |
| Trimester 47 | -0.633*** | -0.400*** | -0.484** | -0.695*** | . |
| Trimester 48 | -0.648*** | -0.405*** | -0.491** | -0.706*** | . |
| Trimester 49 | -0.664*** | -0.410*** | -0.500** | -0.742*** | . |
| Trimester 50 | -0.687*** | -0.421*** | -0.505** | -0.750*** | . |
| Trimester 51 | -0.699*** | -0.444*** | -0.511** | -0.771*** | . |
| Trimester 52 | -0.712*** | -0.457*** | -0.518** | -0.801*** | . |
| Trimester 53 | -0.730*** | -0.473*** | -0.519** | -0.800*** | . |
| Trimester 54 | -0.746*** | -0.483*** | -0.521** | -0.781*** | . |
| Trimester 55 | -0.767*** | -0.491*** | -0.523** | -0.790*** | . |
| Trimester 56 | -0.781*** | -0.497*** | -0.528** | -0.800*** | . |
| Trimester 57 | -0.792*** | -0.502*** | -0.536** | -0.803*** | . |
| Trimester 58 | -0.810*** | -0.514*** | -0.538* | -0.806*** | . |
| Trimester 59 | -0.824*** | -0.523*** | -0.539* | -0.809*** | . |
| Trimester 60 | -0.840*** | -0.530*** | -0.540* | -0.817*** | . |
| Trimester 61 | -0.860*** | -0.540*** | -0.542* | -0.826*** | . |
| Trimester 62 | -0.880*** | -0.542*** | -0.548* | -0.834*** | . |
| Trimester 63 | -0.915*** | -0.557*** | -0.563* | -0.841*** | . |
| Trimester 64 | -0.937*** | -0.566*** | -0.564* | -0.832*** | . |
| Trimester 65 | -0.958*** | -0.573*** | -0.573* | -0.838*** | . |
| Trimester 66 | -0.981*** | -0.583*** | -0.583* | -0.849*** | . |
| Trimester 67 | -1.010*** | -0.622*** | -0.591* | -0.861*** | . |
| Trimester 68 | -1.027*** | -0.640*** | -0.599* | -0.863*** | . |
| Trimester 69 | -1.046*** | -0.651*** | -0.605* | -0.865*** | . |
| Trimester 70 | -1.065*** | -0.661*** | -0.612* | -0.866*** | . |
| Trimester 71 | -1.088*** | -0.671*** | -0.617* | -0.888*** | . |
| Trimester 72 | -1.107*** | -0.681*** | -0.621* | . | . |
| Trimester 73 | -1.137*** | -0.692*** | -0.623* | . | . |
| Trimester 74 | -1.183*** | -0.702*** | -0.624* | . | . |
| Trimester 75 | -1.197*** | -0.706*** | -0.622* | . | . |
| Trimester 76 | -1.205*** | -0.687*** | -0.629* | . | . |
| Trimester 77 | -1.235*** | -0.664** | -0.647* | . | . |
| Trimester 78 | -1.252*** | -0.676** | -0.649* | . | . |
| Trimester 79 | -1.249*** | -0.664** | -0.647* | . | . |
| Trimester 80 | -1.270*** | -0.653** | -0.605 | . | . |
| Trimester 81 | -1.303*** | -0.665** | -0.583 | . | . |
| Trimester 82 | -1.331*** | -0.675** | -0.581 | . | . |
| Trimester 83 | -1.362*** | -0.703** | -0.642 | . | . |
| Trimester 84 | -1.384*** | -0.712** | -0.628 | . | . |
| Trimester 85 | -1.380** | -0.721** | -0.546 | . | . |
| Trimester 86 | -1.363** | -0.782*** | -0.546 | . | . |
| Trimester 87 | -1.364** | -0.778** | . | . | . |
| Trimester 88 | -1.313** | -0.806** | . | . | . |
| Trimester 89 | -1.222** | -1.013** | . | . | . |
| Trimester 90 | -1.305** | . | . | . | . |
| Age | -0.00584 | -0.00301 | 0.0208*** | 0.0157 | -0.0326 |
| Age squared | 0.000015 | 0.0000068 | -0.000016*** | -0.00001 | 0.000023 |
| *Education (ref: low)* |  |  |  |  |  |
| Low intermediate | 0.0595 | 0.0829 | 0.0672 | 0.00846 | 0.0600*** |
| High intermediate | 0.0318 | -4.382 | -0.089 | 0.209*** | . |
| High | 0.101 | -3.732 | -0.0206 | . | 0.530*** |
| Career quality before unemployment | 0.166** | 0.257*** | 0.167 | 0.360** | 0.105*** |
| GDP | 0.0104 | -0.00344 | -0.00556 | -0.000971 | -0.00189 |
| Constant | 0.0346 | -38.13*** | -18.44*** | -7.429 | 11.26 |
| Observations | 16,545 | 19,233 | 8,639 | 4,631 | 2,629 |

Note: grayed cells indicate that effects are significantly different (p<0.05) compared to the reference category. This was tested in a model including interactions between each age group and time, with the youngest age group as reference category.
