# Supplementary material for: The cumulative disadvantage of unemployment: Longitudinal evidence across gender and age at first unemployment in Germany
Source: PLoS One. 2020 Jun 24;15(6):e0234786. doi: 10.1371/journal.pone.0234786 (PMC7313743; doi:10.1371/journal.pone.0234786)
Supplement: Attachment — Submitted filename: Review.docx [file pone.0234786.s004.docx]

The study uses detailed employment data from the German Socio-Economic Panel Study over a long period of time after people’s first unemployment experience.

The study is very original in a three important aspects: (1) the long follow-up period after unemployment that is usually not considered in other research, (2) the measure of career quality as a time-changing variable that takes the labour market experiences since unemployment into account (most other studies focus just on wage at a point in time), (3) the focus on first unemployment whereas most other studies do not account for previous unemployment experience. Furthermore, I find it applaudable that gender and age differences are explicitly considered even thought I have some issues with respect to the research design you use to examine these gender-age variations (see below). However, I see a few fundamental problems with the research design of the paper in its current form. Furthermore, I have some other points with respect to the theoretical and methodological part of the paper. .

**Fundamental problems**

Hypothesis 1A and H1B state that the career quality of previously unemployed individuals will either decrease or increase over the post-unemployment period. However, it seems that this increase is measured not in comparison to the career quality prior to unemployment but in comparison to the state of unemployment itself (trimester 0). That makes H1A an impossible scenario, the career quality cannot further decrease compared to being unemployed.

There is an inconsistency in H2B and H3. Both hypotheses combined do not allow for variation in the effect of age for men. If the age groups 25-45 (H2b) and older workers (45+) (H3) all have slower expected recovery after unemployment then there is no age group that does not have slower expected recovery. Or did you mean workers aged 18-25 should have less strong unemployment scars? Also this is a problematic assertion because it (1) is inconsistent with previous literature as larger unemployment scars have been found for unemployment experienced at younger age groups in previous research and (2) the age group 18-25 is not represented in all labour market segments because the highly educated group is still studying in that age bracket in Germany.

I miss a comparison group of the people who do not experience unemployment. Therefore, your unexpected gender finding that contradicts previous research could be due to the fact that also women who do not experience unemployment are more likely than men to interrupt their careers and have spells of inactivity and non-work. This is a fundamental design problem of your study.

**Other points**

The theoretical part of the text is not always consistent. You start with elaborating on three reasons for labour market disadvantage after unemployment: human capital depreciation, signaling and labour market segmentation (p.6-7). The third theory is quite different in nature than the first two and this needs to be acknowledged. While the first two theories listed -human capital depreciation and signaling- are indeed explaining why the experience of unemployment would lead to worse outcomes, the labour market segmentation theory is merely stating that there are jobs with worse conditions in the economy but does not at all explain the reasons for unemployment scarring. To the contrary, labour market segmentation means that some jobs are more at risk for unemployment to begin with and hence the effect of unemployment is endogenous. Part of the issue is also that the first two theories are micro-theories while the segmentation theory is a macro theory. I suggest that the authors reconsider carefully how to build up the theoretical argument.

Furthermore, the human capital depreciation theory and signaling theory are often just mentioned as possible explanations for labour market disadvantage after unemployment without being explicitly tested. It would be good if you could indicate in the review of previous research evidence which previous studies actually found evidence for the mechanism and which studies merely mentioned the mechanism as a possible explanation without any attempt to test it.

It is unclear whether you managed to single out the *first* unemployment experience only for those respondents who had their first interview at age 18 or also for respondents who had their first interview at a later age. See last sentence page 11 and first sentences page 12 (line 251 onwards): but who retrospectively reported to have been continuously employed (either with the same or another employer) since they were 18 ***at their first interview***

If you look at the first unemployment experience for all age groups then this should be reflected in the theoretical part and the hypotheses. Because unemployment is a different experience if it is a first time experience or a recurrent experience and it is unclear from your overview what the results of previous studies refer to.

While I like the concept and measure of career quality and I think it is highly original, it is a pity that all states of employment are treated as having the best level of career quality. You mention in your paper the large differentiation in types of jobs and labour market segmentation but that is an aspect that you’re not taking into account in the measure of career quality. Perhaps you could extend the measure by differentiating between types of employment?

Period effects. While you mention that you exclude the period of the most recent recession and you control for the year’s GDP/capita, it would be good to provide some information about ***how*** the economic climate might have affected the age of first unemployment experience and how this could have affected your results.
