# Supplementary material for: The cumulative disadvantage of unemployment: Longitudinal evidence across gender and age at first unemployment in Germany
Source: PLoS One. 2020 Jun 24;15(6):e0234786. doi: 10.1371/journal.pone.0234786 (PMC7313743; doi:10.1371/journal.pone.0234786)
Supplement: Attachment — Submitted filename: Response to Reviewers.docx [file pone.0234786.s005.docx]

**Response to Reviewer 1**

Thank you for the positive comments on our manuscript. We are glad to hear that – with exception of two final issues - we addressed all your concerns both appropriately and rigorously. We are grateful for the very useful feedback and suggestions, which helped us further improve our work. Please find below a set of notes summarizing our responses to your comments. Your original comments are reproduced in italics font, while our answers are preceded by “A”.

*I fail to see why the post-recessionary period is left out of the analysis. I find it difficult to accept that "stable economic conditions" is a requirement for these types of analyses. Economic busts and booms is a major part of the labor market in capitalistic societies, and it seems weird to just drop these observational points from the analysis altogether. At the very least, you should run the analysis for the pre- and post-recessionary period separately, and compare and discuss the differences and similarities. In fact, this would probably strengthen the study immensely and make it considerably more interesting.*

A: In what follows, we provide additional evidence refuting a pre/post 2005 type of analysis. We start by including 10 additional waves of the GSOEP data extending our sample to 2015. Next, we estimate a series of fixed-effect regression panel models predicting the effect of time since first unemployment on career quality, separately across two periods. That is, 10 years before and 10 years after 2005. The coefficient estimates for the effect of time since first unemployment on the level of career quality across these two periods are shown in Figure 1 below. In addition, we also estimate a model that merges both periods before and after 2005 and controls for the time pre/post 2005. This reflects the size of the bias in the coefficients had we included data post 2005.

*Figure 1. Pre/Post Analysis*

Figure 1 reveals two important findings. First, effects of time since initial unemployment on career quality are considerably greater after 2005 than before 2005. For example, in the first year after unemployment the size of the coefficient in the post 2005 model is double the size of the coefficients in the pre 2005 model. We observe major differences in the coefficient estimates pre/post 2005 over a 10-year post unemployment time span (as evidenced by non-overlapping confidence intervals). Second, the recovery from unemployment post 2005 is considerably slower. These two key results reflect our concern that extending the analyses beyond 2005 will inflate and thereby bias our coefficient estimates downwards.

One way to account for this bias, would be to apply a difference-in-difference approach. However, because the recession affected all workers, we are left with no control group to permit such an approach. The absence of a proper control and treatment group (in addition to a period before/after 2005) prevents us from using a difference-in-difference design to separate out recession-specific from individual-specific effects. The approach that you are suggesting – i.e., running separate analyses in the period pre/post 2005 and discussing similarities and differences in the two periods – is problematic for another reason.

Substantively, it would generate a fundamentally different paper addressing different research questions compared to the ones we set up to address. In our original manuscript, we did discuss the issue around the observation window (i.e., lines 639-641), listing this as a potential avenue for future research. Overall, we believe that we have provided both empirical evidence and substantive arguments to justify our decision to investigate careers over a 22 year period (instead of extending analyses beyond 2005). We hope that you will be sympathetic to our efforts to respond diligently to your comments.

*It is still unclear exactly how and why the current analytical strategy is better than the alternatives. The study would actually profit from including a 'traditional' analysis (after first specifying what the traditional and poorer alternatives are) directly, and comparing the empirical results with the 'new' analytical strategy that the authors prefer. What is gained, and what is lost, with the new approach?*

A: Previous studies on the effects of unemployment – which we assume you refer to as “traditional approaches” have usually focussed on one unemployment dimension (i.e., occurrence or spell of previous unemployment) on outcomes such as earnings, employment, health or fertility of displaced workers. While valuable, this approach fails to capture labor force status changes over the course of one’s career, and how these in turn shape people’s employment trajectories and their level of career success in the future. Research on career ‘turbulence’ or ‘complexity’ based on a sequence analytical approach exist, but these measure the ‘volatility’ of labor force changes rather than the quality of these changes.

In order to permit analysis of the degree and speed of recovery from an initial unemployment experience, we believe that a holistic perspective is necessary, which accounts for multiple dimensions of unemployment (e.g., occurrence, duration, frequency) simultaneously. As we outline in the Introduction, our sequence analytic approach provides such a holistic and multidimensional perspective. It quantifies the quality of labor force status trajectories in a dynamic way, capturing the volatility of labor force trajectories, their evolution since the occurrence of an initial unemployment spell and most importantly the quality of these transitions. In doing so, it advances “traditional” approaches that are based on instantaneous measures and a single dimension of unemployment. We also believe that by quantifying the quality of career success we make an important contribution to the sequence analytical approach. These issues are discussed and summarized in lines 142-158 of our manuscript. Nevertheless, following your advice we have now revised the Introduction and discuss the knowledge gaps more clearly (i.e., lines 71-79).

Thank you very much for your helpful suggestions and encouragement! We hope that we have been able to address your concerns.
